# Effects of Pomegranate Juice Supplementation on Oxidative Stress Biomarkers Following Weightlifting Exercise

**DOI:** 10.3390/nu9080819

**Published:** 2017-07-29

**Authors:** Achraf Ammar, Mouna Turki, Omar Hammouda, Hamdi Chtourou, Khaled Trabelsi, Mohamed Bouaziz, Osama Abdelkarim, Anita Hoekelmann, Fatma Ayadi, Nizar Souissi, Stephen J. Bailey, Tarak Driss, Sourour Yaich

**Affiliations:** 1 Research Unit: Education, Motricity, Sport and health, UR15JS01, High Institute of Sport and Physical Education of Sfax, Sfax University, Sfax 3000, Tunisia; hammouda.o@parisnanterre.fr (O.H.); h_chtourou@yahoo.fr (H.C.); trabelsikhaled@gmail.com (K.T.); 2 Institute of Sport Science, Otto-von-Guericke-University, Magdeburg 39106, Germany; osamahalim@ymail.com (O.A.); anita.hoekelmann@ovgu.de (A.H.); 3 Laboratory of Biochemistry, CHU Habib Bourguiba, Sfax University, Sfax 3000, Tunisia; mouna.turki@gmail.com (M.T.); ayadi_fatma@medecinesfax.org (F.A.); 4 Research Center on Sport and Movement (Centre de Recherches sur le Sport et le Mouvement, CeRSM), UPL, Univ Paris Nanterre, UFR STAPS, Nanterre F-92000, France; tarak.driss@parisnanterre.fr; 5 High Institute of Biotechnology, Sfax University, Sfax 3000, Tunisia; mohamed.bouaziz@cbs.rnrt.tn; 6 National Observatory of Sport, Tunis 1003, Tunisia; n_souissi@yahoo.fr; 7 School of Sport, Exercise and Health Sciences, Loughborough University, Loughborough LE11 3AJ, UK; S.Bailey2@lboro.ac.uk; 8 Department of Community Medicine and Epidemiology, Hédi Chaker Hospital, Sfax University, Sfax 3000, Tunisia; kammoun.sourour@laposte.net

**Keywords:** lipid peroxidation, power training, polyphenol, antioxidant

## Abstract

The aim of this study was to test the hypothesis that pomegranate juice supplementation would blunt acute and delayed oxidative stress responses after a weightlifting training session. Nine elite weightlifters (21.0 ± 1 years) performed two Olympic-Weightlifting sessions after ingesting either the placebo or pomegranate juice supplements. Venous blood samples were collected at rest and 3 min and 48 h after each session. Compared to the placebo condition, pomegranate juice supplementation attenuated the increase in malondialdehyde (−12.5%; *p* < 0.01) and enhanced the enzymatic (+8.6% for catalase and +6.8% for glutathione peroxidase; *p* < 0.05) and non-enzymatic (+12.6% for uric acid and +5.7% for total bilirubin; *p* < 0.01) antioxidant responses shortly (3 min) after completion of the training session. Additionally, during the 48 h recovery period, pomegranate juice supplementation accelerated (*p* < 0.05) the recovery kinetics of the malondialdehyde (5.6%) and the enzymatic antioxidant defenses compared to the placebo condition (9 to 10%). In conclusion, supplementation with pomegranate juice has the potential to attenuate oxidative stress by enhancing antioxidant responses assessed acutely and up to 48 h following an intensive weightlifting training session. Therefore, elite weightlifters might benefit from blunted oxidative stress responses following intensive weightlifting sessions, which could have implications for recovery between training sessions.

## 1. Introduction

Oxidative stress reflects an imbalance between oxidant production and antioxidant responses where the former exceeds the latter. It is well documented that strenuous exercise acutely increases oxidative stress biomarkers and is accompanied by a prolonged pro-oxidant redox status following such exercise [1,2,3,4]. Indeed, it has been reported that lipid peroxidation markers are increased immediately following exercise performed near the anaerobic threshold intensity [5], and during short-term maximal efforts such as sprint [6,7] and strength exercises [3,4,8], with such redox perturbations maintained for up to 48 h following high intensity exercise [2,3,4]. These changes in oxidative stress biomarkers are also accompanied by an increase in antioxidant responses following exercise. Specifically, intensive physical efforts (i.e., sprint, swimming, strength, and Wingate exercises) have been shown to increase the content of glutathione peroxidase (GPX), catalase (CAT), uric acid (UA), and total bilirubin (Tbil) immediately post exercise [3,9,10,11] and to increase the content of CAT and GPX up to 48 h after weightlifting exercise [2,3,4].

On the other hand, there is emerging evidence that supplementation with pomegranate juice (POMj), which is a rich source of polyphenols and other biologically active compounds (e.g., flavonols, flavanoids, gallicacid, ellagic acid, quercetin, ellagitannins, and nitrate) [12,13], confers several health benefits during stressful situations [14]. Indeed, POMj has been shown to prevent oxidative stress by enhancing antioxidant status (+130%) [15] and reducing oxidative stress biomarkers including, lipid peroxidation (−65%) and low-density lipoprotein oxidation (−90%) [16]. Additionally, POMj has been reported to exhibit the most powerful antioxidant effect compared to other fruit and vegetable juices [17,18]. Indeed, after a comparative spectrophotometric study, POMj was shown to be the most effective in reducing low-density lipoprotein oxidation and inhibiting cellular oxidative stress in macrophages compared to green tea, red wine, and orange, blueberry and cranberry juices [17]. Similarly, using an in vitro comparative assay, POMj manifested the highest capacity to neutralize free radicals with an antioxidant activity three times higher than red wine and green tea (Trolox equivalent antioxidant capacity = 18–20 vs. 6–8) [18]. The underlying mechanisms mediating the potent antioxidant properties of POMj are not yet clear, but its effectiveness has been attributed to enhanced polyphenol bioavailability compared to other foods rich in polyphenols [17].

Although it is established that polyphenol-rich POMj can improve numerous physiological processes in individuals placed under stress [14,15,16] and that physical exercise is a considerable physiological stressor [1,2,3,4,19], relatively few studies have evaluated the antioxidant potential of POMj supplementation during and following intense physical activity [20,21,22]. Moreover, despite evidence that Olympic weightlifting exercises are often accompanied by an acute and delayed increase in oxidative stress biomarkers [2,3,4,8], only one recent study has evaluated the efficacy of POMj supplementation to modulate biochemical responses (i.e., inflammatory and muscle damage parameters) during a weightlifting exercise session [23]. However, biomarkers of antioxidant capacity and oxidative stress, which are considered as key components of muscle fatigue and overtraining syndrome [1], were not measured in this study. Therefore, further research is required to assess the potential biochemical basis for POMj as a nutritional recovery strategy post intensive weightlifting exercise.

The aim of the present study was to assess whether consumption of natural POMj could reduce the immediate increase of oxidative stress responses and accelerate the recovery of such responses following a weightlifting training session. We hypothesized that the consumption of natural POMj could blunt acute and delayed oxidative stress responses following a weightlifting training session, which may help in recovering the resting levels of redox parameters at 48 h post exercises.

## 2. Materials and Methods

### 2.1. Participants

Nine elite male weightlifters (21 ± 1years, 80 ± 10kg, 1.75 ± 0.08m (mean ± standard deviation (SD))) volunteered to participate in this study. The participants were recruited on the basis of: (i) they trained at least five sessions per week, (ii) they had at least 3 years’ experience of Olympic weightlifting,(iii) they did not have any injuries, and (iv) they did not use any antioxidant or anti-inflammatory drugs during the experimental period nor for one month before commencement of the study [23]. Additionally, participants were instructed to avoid the consumption of creatine and the consumption of large amounts of foods rich in antioxidants or polyphenols (e.g., blueberries, coffee, tea, grape, cherry, curcuma, red wine, and dark chocolate [24]) and they self-reported that they adhered to this requirement. After receiving a thorough explanation of the possible risks and discomforts associated with the experimental procedures, each participant provided their written informed consent to take part in the experiment. The study was conducted according to the Declaration of Helsinki. The protocol and the consent form were fully approved (identification code: 8/16) by the review board “Local committee of the Laboratory of Biochemistry, CHU Habib Bourguiba, Sfax, Tunisia” before the commencement of the assessments. Additionally, all ongoing and related trials for this intervention are registered in Clinical Trials.gov (identification code: NCT02697903).

### 2.2. Experimental Design

**Figure 1:**
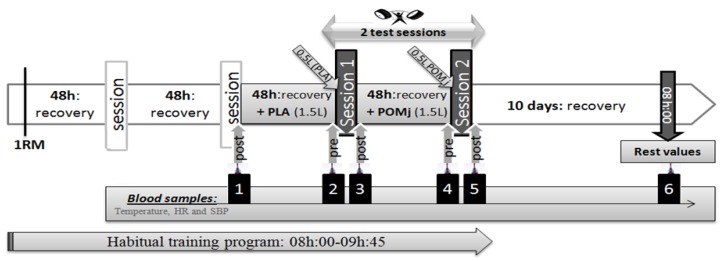
Experimental design.1-RM, One-repetition maximum; PLA, placebo; POMj, pomegranate juice.

One week before the start of the experimental period, the heaviest weight lifted in a single repetition (1-RM) was assessed for each participant in each Olympic movement (Figure 1). Thereafter, participants performed, as part of their habitual training-program from 08:00 to 09:45, two training sessions (Figure 1) after ingesting the placebo (PLA) (before session 1) and POMj (before session 2) supplements. A recovery period of 48 h separated the PLA and POMj training sessions. Each training session comprised three Olympic Weightlifting exercises (snatch, clean and jerk, and squat) with five sets for each exercise. Specifically, participants completed 2 sets of 3 repetitions at 85% of 1-RM and 3 sets of 2 repetitions at 90% of 1-RM [3,4,23,25]. The PLA and POMj supplements were administered in 250 mL doses and ingested three times daily during the 48 h preceding these two training sessions. Moreover, to allow sufficient time for circulating polyphenols to become elevated and to fully exercise their antioxidant and ergogenic effects [20,21,22,23], participants consumed an additional 500mL of PLA or POMj 60 min before the training sessions (Figure 1) [23]. Before and after each training session, fasting blood samples (blood samples 2–5, Figure 1) were collected. Additionally, to assess the recovery kinetics of the biological parameters, blood samples were collected in a resting recovered state (i.e., after 10 days of recovery (blood sample 6) and immediately (3 min) after the training session which preceded the PLA session (blood sample 1) [23].

Before test sessions, participants underwent an overnight fast and were only permitted to drink one glass of water (15–20 cL) to avoid the potential confounding influence of postprandial thermogenesis [23,26]. Additionally, given that randomly assigning the supplements would have resulted in some participants consuming the POMj supplement before the PLA supplementation with only a 48 h wash-out period, and given that the beneficial effects of POMj could persist for up to three weeks after consumption [27], we elected to avoid any alteration in the biochemical blood levels at the beginning and during the PLA administration period by avoiding the consumption of POMj before PLA. Therefore, as previously described by Ammar et al. [23], PLA was administered before receiving the POMj supplement.

### 2.3. Pomegranate Juice and Placebo Supplementations

The natural POMj was prepared from a fresh pomegranate fruit 48 h before the beginning of the experimentation and was frozen and stored at −4 °C. No additional chemical products were added to the natural POMj. Each 500 mL of POMj contained 2.56 g of total polyphenols, 1.08 g of orthodiphenols, 292.6 mg of flavonoids, and 46.75 mg of flavonols [23]. PLA juice consisted of a pomegranate-flavored commercial drink containing water, citric acid, natural flavor and natural identical flavor (pomegranate), sweeteners (aspartame × (0.3 g/L), acesulfameK (0.16 g/L), stabilizers (Arabic-gum), and did not contain antioxidants, vitamins, nor polyphenols [23].

### 2.4. Phenolic Compounds

#### 2.4.1. Extraction of Phenolic Fraction

The phenolic extracts were obtained following the procedure of Chtourou et al. [28] with some modifications. Firstly, the POMj sample (4 g) was added to 2 mL of *n*-hexane and 4 mL of a methanol/water (60:40, *v*/*v*) mixture in a 20 mL centrifuge tube. After vigorous mixing, they were centrifuged for 3 min. The hydroalcoholic phase was collected, and the hexane phase was re-extracted twice with 4 mL of the methanol/water (60:40, *v*/*v*) solution each time. Finally, the hydro alcoholic fractions were combined, washed with 4 mL of *n*-hexane to remove the residual POMj, then concentrated and dried by evaporative centrifuge in vacuum at 35 °C.

#### 2.4.2. Determination of the Total Phenol and *O*-Diphenol Contents

The determination of the total phenolic compounds was performed by means of the Folin-Ciocalteau reagent using the method described by Gargouri et al. [29]. The total phenolic content was expressed as milligrams of gallic acid (GA) equivalent per kilogram of pomegranate (POM) (y = 0.011x, R^2^ = 0.990). The optical density (OD) was measured at λ = 765 nm using a spectrophotometer (Shimadzu UV-1800 PC, Shimadzu, Kyoto, Japan). The concentration of *o*-diphenolic compounds in the methanolic extract was determined by the method of Dridi-Gargouri et al. [30]. The total *o*-diphenolic content was expressed as milligrams of GA equivalent per kilogram of POM (y = 1.144x, R^2^ = 0.999). The OD was measured at λ = 370 nm, using the same spectrophotometer.

#### 2.4.3. Determination of Total Flavonoids

Total flavonoids were measured by a colorimetric assay developed by Gargouri et al. [29]; 1 mL aliquot of appropriately diluted sample or standard solutions of catechin (20, 40, 60, 80, and 100 mg/L) was added to a 10mL volumetric flask containing 4 mL double-distillate H_2_O. At zero time, 0.30 mL 5% Sodium nitrite (NaNO_2_) was added to the flask. After 5 min, 0.30 mL 10% Aluminium chloride (AlCl_3_) was added. At 6 min, 2 mL (1 mol·L^−1^) NaOH was added to the mixture. Immediately, the reaction flask was diluted to volume with the addition of 2.40 mL of double-distillate H_2_O and thoroughly mixed. Concerning the absorbance of the mixture, pink in color, it was determined at 510 nm versus prepared water blank. As for the total flavonoids of fruits, they were expressed on a fresh weight basis as mg/100 g catechin equivalents. It is worth noting that the samples were analyzed in triplicate.

### 2.5. Blood Sampling and Analysis

Blood samples (7 mL) were collected from a forearm vein. Samples were placed in an ice bath and immediately centrifuged at 2500 rpm and 4 °C for 10 min. Aliquots of the resulting plasma were stored at −80 °C until analysis. To eliminate inter-assay-variance, all samples were analyzed in the same assay run. All assays were performed in-duplicate in the same laboratory with simultaneous use of a control serum from Randox. UA and Tbil were determined spectrophotometrically (Architect Ci-4100-ABBOTT, Abbott Deutschland, Wiesbaden, Germany) using uricase and diazonium methods, respectively, and using commercial kits (Ref AU: 3P39-21, Ref Tbil: 6L45, Abbott Deutschland, Wiesbaden, Germany) with an intra-assay-coefficient of variation of 0.5% [3]. CAT activity was measured by assessing the decrease in H_2_O_2_ concentration [31]. When H_2_O_2_ was added at low concentration (0.2 M) to a sample with CAT, this enzyme catalyzed the transformation of this substrate to oxygen and water. To check the activity, a kinetic curve had to be measured for 30 s at λ = 240 nm using a molar extinction coefficient of 43.6 cm^−1^M^−1^ to know the amount of H_2_O_2_ eliminated [31]. To determine GPX activity, the continuous decrease in nicotinamide adenine dinucleotide phosphate (NADPH) concentration was measured, while glutathione (GSH) levels were maintained, following the methods described by Flohe and Gunzler [32]. This method is based on the rise of the absorbance, during 3 min at λ = 340 nm, because of the oxidation of NADPH in presence of GSH, t-butyl hydroperoxide, glutathione reductase (GR), and the sample. The molar extinction coefficient used for the calculations was *ε* = 6.22 × 10^3^ cm^−1^M^−1^. One CAT or GPX enzymatic unit (units) was defined as the amount of the enzyme that catalyzes the conversion of one micromole of substrate per minute. Malonaldehyde (MDA) was measured according to procedures described by Wong et al. [33]. Plasma proteins were precipitated with methanol and removed from the reaction mixture by centrifugation. The protein-free extract was fractionated by high pressure liquid chromatography (HPLC) on a column of octadecyl silica gel, to separate the MDA-thiobarbituric acid (TBA) adduct from interfering chromogens. The MDA-TBA adduct was eluted from the column with methanol/phosphate buffer and quantified spectrophotometrically at λ = 532 nm. Plasma lipoperoxide concentrations were computed by reference to a calibration curve prepared by assays of tetraethoxypropane.

### 2.6. Statistical Analyses

All statistical analyses were performed using STATISTICA 10.0 Software (StatSoft, Maisons-Alfort, France). Normality of the data distribution was confirmed using the Shapiro-Wilks-W-test. To analyze the effect of POMj supplementation on the biological responses during training sessions (pre-post values), a two-way analysis of variance ANOVA (2 levels (supplementation (PLA and POMj)) × 2 levels (training-session (Pre and Post)) with repeated measures) was employed. To analyze the effect of POMj supplementation on the recovery kinetics of the selected parameters, a one-way ANOVA was utilized. When significant main effects were observed, Tukey’s honest-significance-difference (HSD) post-hoc tests were conducted. Effect sizes were calculated as partial eta-squared (ηp^2^) for the ANOVA analysis to assess the potential practical significance of the findings. Pearson correlation was used to assess the correlation between the responses of MDA and the antioxidant markers. Statistical significance was set at *p* < 0.05 and data are presented as mean ± SD unless otherwise stated.

## 3. Results

### 3.1. Acute Effect of POMj on Oxidative Stress Biomarkers Following a Weightlifting Training Session

The oxidative stress responses immediately (3 min) after the weightlifting training sessions are presented in Table 1. The lipid peroxidation biomarker, MDA, increased pre-post training sessions during both PLA (*p* = 0.0002) and POMj conditions (*p* = 0.0006). However, the rate of increase was lower (−12.47%) after POMj supplementation compared to that of PLA supplementation (*p* < 0.01). Similarly, biomarkers of enzymatic and non-enzymatic antioxidants increased pre to post training session completed with both the PLA (*p* = 0.04 for CAT, and GPX and *p* = 0.02 for UA and Tbil) and POMj (*p* = 0.0004 for GPX, *p* = 0.0003 for CAT, and Tbil and *p* = 0.0001 for UA) supplements. However, the rates of increase pre/post training session were enhanced after POMj supplementation (+8.59%, +6.76%, +12.63%, and +5.68% for CAT, GPX, UA, and Tbil, respectively). For all these parameters (i.e., MDA, CAT, GPX, UA, and Tbil), a significant training-session (*p* = 0.003, *p* = 0.009, *p* = 0.008, *p* = 0.005, and *p* = 0.006, respectively) and POMj (*p* = 0.004, *p* = 0.008, *p* = 0.03, *p* = 0.006, and *p* = 0.04, respectively) effects were observed. Additionally, significant interactions (Training session × supplementation condition) were observed for MDA (*p* = 0.02), CAT (*p* = 0.04), and UA (*p* = 0.03).

### 3.2. Delayed Effect of POMj on the Recovery Kinetics of the Oxidative Stress Parameters

Table 2 shows the values of the oxidative stress parameters immediately (3 min), 48 h, and 10 days (resting values) after the training sessions during the PLA and POMj trials. From 3 min to 48 h after the training session, the markers of lipid peroxidation as well as the markers of antioxidant responses decreased significantly in both PLA (*p* = 0.004 for MDA, *p* = 0.008 for CAT and GPX, *p* = 0.005 for UA, and *p* = 0.007 for Tbil) and POMj (*p* = 0.002 for MDA, *p* = 0.004 for CAT and GPX, and *p* = 0.003 for UA and Tbil) conditions. However, the rates of decrease were higher (*p* < 0.05) following POMj supplementation compared to that of PLA supplementation (i.e., Δ rate of decrease = 5.63%, 8.94%, 10.21%, 3.57%, and 7.42% for MDA, CAT, GPX, UA, and Tbil, respectively).

Moreover, after supplementing with POMj, the 48 h recovery period was sufficient to recover the resting values of all parameters (i.e., no significant differences were observed between the values of 48 h recovery and the rest values, *p* > 0.05). However, following PLA supplementation, the resting values of the non-enzymatic antioxidants (i.e., UA and Tbil) were recovered after 48 h, but the values of MDA and the enzymatic antioxidants (i.e., CAT and GPX) remained elevated after the same period (*p* = 0.03 for MDA and *p* = 0.02 for CAT and GPX, between the values at 48 h and at rest).

### 3.3. Relationship between the Responses of Lipid Peroxidation and Antioxidant System

Table 3 shows the relationship between the responses (Δ% rate of change from PLA to POMj conditions) of MDA and the antioxidant parameters. Regression lines for significant correlations were plotted and added to the data supplement (Figure S1 and S2). The acute MDA content following weightlifting exercise was significantly correlated (Table 3, Figure S1) to the enzymatic (*r* = 0.6, *p* = 0.005 with CAT and *r* = 0.5, *p* = 0.03 with GPX) and non-enzymatic antioxidant markers with the highest correlation registered between MDA and UA (*r* = 0.7, *p* = 0.0008). With regard to the delayed response of MDA during the 48 h recovery period, the change in the rate of lipid peroxidation was only correlated (Table 3, Figure S2) to the enzymatic antioxidants CAT (*r* = 0.6, *p* = 0.006) and GPX (*r* = 0.5, *p* = 0.02).

## 4. Discussion and conclusions

The aim of the present study was to investigate the effect of supplementation with a natural POMj on the acute and delayed oxidative stress responses following a weightlifting training session. The main results showed that, compared to PLA, the consumption of POMj 48 h before and during the training session enhanced the recovery kinetics of the acute and delayed oxidative stress responses. Specifically, there was: (i) a reduction in the immediate increase of the MDA (−12.47%), (ii) an increase in acute antioxidant responses (e.g., +12.63% and +8.59% for UA and CAT, respectively), and (iii) an acceleration in the delayed recovery kinetics of MDA (5.63%) and the antioxidant markers (e.g., 8.94% for CAT and 10.21% for GPX) with POMj compared to that of PLA supplementation.

### 4.1. Effect of Weightlifting Training Sessions on Oxidative Stress Responses

The present study reported a significant training-session effect on the oxidative stress parameters (i.e., lipid peroxidation and antioxidant markers) with higher values following a training session compared to pre-training session values in both experimental conditions. These findings are in line with previous studies reporting increased oxidative stress biomarkers following weightlifting exercises [3,4,8]. Exercise-induced oxidative stress has been attributed to reactive oxygen species (ROS) generated through the enzymes xanthine oxidase, NADPH oxidase, and phospholipase A2 [19]. For example, xanthine oxidase has been implicated as an important contributor to oxidative injury to tissues [34], especially during exercise [1] and after ischemic insults [35]. Moreover, increases in hydrogen ions (H^+^), catecholamine production, as well as muscle damage, and inflammation post-intensive exercise have been also suggested to contribute to the increased oxidative stress [1,3,7,34]. These effects might be linked to leucocyte adhesion to the endothelial wall [36]; excessive nitric oxide production in activated macrophages [36], which would be expected to facilitate the production of the potent oxidizing and nitrating species peroxynitrite in an environment of increased superoxide production; and iron liberation from haemoglobin or ferritin, promoting Fenton chemistry and the generation of the potent oxidizing hydroxyl radicals [37,38]. These factors are likely to have interacted to evoke the exercise-induced oxidative stress observed in the current study, albeit not directly measured currently.

### 4.2. Acute Effect of POMj on Oxidative Stress Responses Following theTraining Session.

In this study, POMj supplementation showed a significant impact on the acute post-exercise MDA and enzymatic and non-enzymatic antioxidant responses. Specifically, a lower rate of increase in MDA and greater rate of increase in antioxidant markers were observed immediately post training in the POMj trial compared to the PLA trial. Previous studies in sedentary participants have demonstrated a potent antioxidant effect of POMj on neutralizing reactive oxygen species (ROS) [15,16,17,18,39]. However, the results of the current study extend these observations to trained subjects and confirm a beneficial effect in attenuating oxidative stress responses provoked by exhaustive physical exercise. These findings are in line with previous results by Mazani et al. [21] who showed that 240mL of POMj consumed daily for 14 days prior to treadmill running exercise (70% max heart rate) significantly increased the activity of enzymatic antioxidants (i.e., higher pre-post exercise change for GPX and superoxide dismutase (SOD) and attenuated markers of lipid peroxidation post exercise. Similarly, these findings are consistent with those of Tsang et al. [22] who showed that one week of POMj consumption (500 mL/day containing 1.69 g total phenolics/L) significantly decreased urinary levels of lipid peroxidation in the POMj group 30 min after treadmill exercise sessions (50% Wmax). The acute attenuation in post-exercise lipid peroxidation responses after POMj supplementation might be linked to the antioxidant properties of POMj [21,22] leading to reduced acute oxidative stress, lipid peroxidation, tissue edema and/or metabolic by-product accumulation [23,40]. Indeed, UA and CAT responses were increased using POMj (12.63% and 8.59%, respectively) and showed the strongest correlation to the acute MDA response (*r* = 0.7 and 0.6, respectively) during the training session in the present study. Therefore, it could be suggested that the antioxidant activities of UA and CAT have the highest effect on reducing the acute MDA response immediately following intensive physical exercise completed after POMj supplementation. The apparent efficacy of POMj as an antioxidant might be attributed to its effectiveness as a polyphenol donor [41].

### 4.3. Delayed Effect of POMj on the Recovery Kinetics of the Oxidative Stress Parameters

After PLA ingestion, only UA and Tbil returned to resting levels 48 h following training with MDA, CAT, and GPX remaining elevated compared to the resting values. These observations confirm previous results showing that a 48 h recovery period lowers MDA levels and antioxidant responses compared to values observed immediately post-training, but was not sufficient to completely recover lipid peroxidation and enzymatic antioxidant parameters to resting levels [3,4]. However, given that the enzymatic (CAT and GPX) but not the non-enzymatic (UA and Tbil) antioxidants remained increased concomitantly with increased markers of lipid peroxidation at 48 h post exercise, and that CAT and GPX correlated with the delayed response of MDA (*r* = 0.6 and *r* = 0.5, respectively), it seems that enzymatic antioxidants have a greater potential to mitigate the delayed oxidizing effects of ROS exposure. In contrast, results from the present study indicate that POMj supplementation during the recovery period facilitated the return of the post-training-session values of all parameters to their resting concentration levels. Indeed, the results showed that the rates of decrease of MDA, CAT, and GPX were higher (5.63%, 8.94%, and 10.21%, respectively) with POMj compared to that of PLA supplementation. The more rapid recovery kinetics of lipid peroxidation and antioxidant markers following high-intensity resistance exercise with POMj supplementation confirms the potent antioxidant properties of polyphenol-rich POMj reported previously [20,27]. Indeed, in healthy non active individuals, 15 days of POMj consumption was shown to enhance antioxidant responses, as evidenced by increased erythrocyte glutathione and serum SOD and GPX content, and reduced MDA, protein carbonyls, and matrix metalloproteinases 2 and 9 levels [21]. Importantly, similar responses (lower MDA and protein carbonyl levels) have also been reported following aerobic-based-exercises in adult endurance athletes [20].

Although the exact mechanisms underlying the antioxidant effects of POMj are not entirely understood [20,21], these effects are likely mediated, at least in part, through the high polyphenol content of POMj [39,42] since polyphenols confer antioxidant effects [41]. Indeed. polyphenols can abate the oxidative damage of proteins, lipids, and other cell constituents by the rapid donation of an electron (accompanied by a hydrogen nucleus) to a free radical (e.g., superoxides, peroxynitrite) from hydroxyl (–OH) groups attached to their phenolic rings [36,43]. Through this scavenging process, polyphenols can chemically reduce and stabilize or inactivate free radical species, thereby inhibiting lipid peroxidation and other oxidative modifications (e.g., low density lipoprotein (LDL) oxidation). Furthermore, the antioxidant effect of polyphenols has been attributed to the suppression of free radical formation by modulating antioxidant enzymes and chelating metal ions (Fe^2+^, Cu^2+^) involved in free radical production [44,45]. Other possible mechanisms that might underpin the antioxidant properties of polyphenols include the inhibition of leucocyte immobilization and xanthine oxidase activity [36], enhanced endothelial function, lowering of the absorption of pro-oxidant nutrients such as iron [46], the modulation of enzymatic activities (i.e., enhancement of glutathione peroxidase, catalase, NADPH-quinoneoxidoreductase, glutathione S-transferase, and/or cytochrome P450 enzyme) [47], and recycling of antioxidant and reducing agents (e.g., vitamin E and C) [41,45]. However, while the present findings offer some insight into some of the underlying antioxidant mechanisms of POMj, more studies are needed to address the mechanisms for elevated UA and CAT post POMj ingestion, how this links to oxidative stress, and what are the tissues (or cells) of action.

Despite our findings suggesting that POMj supplementation has the potential to blunt oxidative stress responses following weightlifting exercise, it has been previously reported that the acute oxidative stress response following such exercise sessions is greater in the morning compared to the evening session (+12%) [3]. Therefore, while the present results showed that POMj supplementation could blunt the morning acute oxidative responses by 12.5% compared to the PLA condition, further research is required to assess whether the effects can be reproduced at different times of day.

In conclusion, the results of the present study extend on previous studies reporting improved protection against oxidative stress in healthy and diseased conditions after POMj consumption by indicating that POMj supplementation can reduce the acute oxidative stress response to an intensive session of resistance exercises by enhancing antioxidant responses and accelerating the recovery kinetics of oxidative stress markers. These findings about reduced oxidative stress following POMj supplementation might have implications for recovery of performance following intensive resistance training.

## 5. Limitations and Perspectives

The present study is the first to demonstrate a potential protective effect of polyphenol-rich POMj supplementation on the degree of lipid peroxidation induced by intense weightlifting exercises, as inferred from a lower circulating MDA content. However, it should be acknowledged that since exercise has been shown to provoke oxidative modifications to several biological components (e.g., proteins, lipids, and DNA) [48], and since MDA is just one product of lipid peroxidation, two or more biomarkers are recommended to more accurately infer oxidative damage [49,50]. Accordingly, further studies using multiple redox-related biomarkers are required to confirm the potential positive effects of POMj supplementation on blunting lipid damage and oxidative stress following physical exercise and to specify the target tissues and the exact antioxidant mechanisms of POMj.

Using the same protocol of the present this study, a recent study reported an increase in the total (+8.3%) and maximal (+3.3%) load lifted during a weightlifting training session, and a lowering in markers of muscle damage and the delayed onset of muscle soreness 48 h after the weightlifting training session after POMj supplementation compared to that of placebo supplementation [23]. Therefore, the findings of the current study might improve understanding of the mechanisms for blunted muscle damage following weightlifting training. However, while consumption of polyphenol-rich beverages can blunt post exercise redox perturbation and muscle damage, and can accelerate the recovery of skeletal muscle force production post strenuous exercise, polyphenol consumption during a training intervention has been reported to blunt some of the physiological adaptations elicited by the training program [51]. Therefore, athletes wishing to use POMj as a potential recovery aid during intensified training periods, where the degree of oxidative stress, inflammation, and muscle damage will be greater, need to balance the aim of promoting recovery from training sessions with the potential to attenuate the exercise-induced redox signaling that provokes physiological adaptations to exercise training. Further research is required to optimize the POMj supplementation guidelines.

## Figures and Tables

**Table 1:** Acute oxidative stress responses to weightlifting training session following pomegranate juice (POMj) and placebo (PLA) supplementation.

Variables	Placebo		% of Change	Pomegranate		% of Change	Δ (POMj-PLA) in %	ANOVA		
	Pre	Post		Pre	Post			Pomegranate Effect	Training Effect	Interaction
**Biomarkers of lipid peroxidation**										
**MDA** (μmol/L)	1.82 ± 0.22	2.44 ± 0.18 *	+34.07%	1.76 ± 0.24	2.15 ± 0.26 *	+22.03%	−12.47%	F_(1,8)_ = 15.9	F_(1,8)_ = 36.8	F_(1,8)_ = 8.4
								*p* = 0.004	*p* = 0.0003,	*p* = 0.02,
								ηp^2^ = 0.3	ηp^2^ = 0.6	ηp^2^ = 0.6
**Biomarkers of enzymatic antioxidant system**										
**CAT** (Units)	15.31 ± 1.51	18.51 ± 1.37 *	+21.02%	15.19 ± 1.32	19.68 ± 1.42 *	+29.61%	+08.59%	F_(1,8)_ = 12.3	F_(1,8)_ = 26.2	F_(1,8)_ = 6.0
								*p* = 0.008	*p* = 0.0009	*p* = 0.04,
								ηp^2^ = 0.5	ηp^2^ = 0.6	ηp^2^ = 0.4
**GPX** (Units)	0.87 ± 0.08	1.04 ± 0.08 *	+20.46%	0.86 ± 0.09	1.09 ± 0.08 *	+27.13%	+06.76%	F_(1,8)_ = 6.9	F_(1,8)_ = 27.3	F_(1,8)_ = 4.3
								*p* = 0.03	*p* = 0.0008	*p* = 0.07
								ηp^2^ = 0.4	ηp^2^ = 0.5	ηp^2^ = 0.2
**Biomarkers of non-enzymatic antioxidant system**										
**UA** (μmol/L)	321.4 ± 19.6	402.3 ± 22.6 *	+25.16%	299.6 ± 16.9	412.8 ± 18.9 *	+37.79%	+12.63%	F_(1,8)_ = 13.72	F_(1,8)_ = 31.6	F_(1,8)_ = 6.9
								*p* = 0.006	*p* = 0.0005	*p* = 0.03
								ηp^2^ = 0.5	ηp^2^ = 0.6	ηp^2^ = 0.6
**Tbil** (μmol/L)	12.30 ± 1.88	15.28 ± 2.32 *	+24.21%	12.01 ± 2.01	15.58 ± 2.43 *	+29.87%	+05.68%	F_(1,8)_ = 5.9	F_(1,8)_ = 29.8	F_(1,8)_ = 3.9
								*p* = 0.04,	*p* = 0.0006	*p* = 0.08
								ηp^2^ = 0.4	ηp^2^ = 0.7	ηp^2^ = 0.1

* Significant differences between pre-post training session. MDA, malonaldehyde; CAT, catalase; GPX, glutathione peroxidase; UA, uric acid; Tbil = total bilirubin; ANOVA, analysis of variance.

**Table 2:** Recovery kinetics of the oxidative stress responses after placebo and pomegranate supplementation.

Variables	3 min Post Training	48 h Recovery	Rest Values (10 days)	Δ 48 h-3 min in %	ANOVA
	PLA				
Biomarkers of lipid peroxidation					
MDA (μmol/L)	2.44 ± 0.18	1.76 ± 0.24 ^a,b^	1.41 ± 0.20	−27.86%	F_(2,16)_ = 12.2, *p* = 0.0006, ηp^2^ = 0.4
Biomarkers of enzymatic antioxidant system					
CAT (Units)	18.51 ± 1.37	15.19 ± 1.32 ^a,b^	13.4 ± 1.14	−17.94%	F_(2,16)_ = 13.3, *p* = 0.0004, ηp^2^ = 0.4
GPX (Units)	1.04 ± 0.08	0.86 ± 0.09 ^a,b^	0.71 ± 0.08	−17.31%	F_(2,16)_ = 12.7, *p* = 0.0005, ηp^2^ = 0.4
Biomarkers of non-enzymatic antioxidant system					
UA (μmol/L)	402.3 ± 22.6	299.6 ± 16.9 ^a^	291.9 ± 18.2	−25.62%	F_(2,16)_ = 5.1, *p* = 0.02, ηp^2^ = 0.5
Tbil (μmol/L)	15.28 ± 2.32	12.01 ± 2.01 ^a^	10.93 ± 1.74	−21.40%	F_(2,16)_ = 4.0, *p* = 0.04, ηp^2^ = 0.5
	**POMj**				
Biomarkers of lipid peroxidation					
MDA (μmol/L)	2.15 ± 0.26	1.43 ± 0.23 ^a^	1.41 ± 0.20	−33.49%	F_(2,16)_ = 6.1, *p* = 0.01, ηp^2^ = 0. 4
Biomarkers of enzymatic antioxidant system					
CAT (Units)	19.68 ± 1.42	14.39 ± 1.44 ^a^	13.4 ± 1.14	−26.88%	F_(2,16)_ = 4.2, *p* = 0.03, ηp^2^ = 0.5
GPX (Units)	1.09 ± 0.08	0.79 ± 0.07 ^a^	0.71 ± 0.08	−27.52%	F_(2,16)_ = 4.4, *p* = 0.03, ηp^2^ = 0.4
Biomarkers of non-enzymatic antioxidant system					
UA (μmol/L)	412.8 ± 18.9	292.3 ± 20.6 ^a^	291.9 ± 18.2	−29.19%	F_(2,16)_ = 3.9, *p* = 0.04, ηp^2^ = 0.6
Tbil (μmol/L)	15.58 ± 2.43	11.09 ± 1.87 ^a^	10.93 ± 1.74	−28.82%	F_(2,16)_ = 3.8, *p* = 0.04, ηp^2^ = 0.5

^a^: Significant differences between 48 h and 3 min post training session. ^b^: Significant difference between 48 h recovery and rest values.

**Table 3:** Relationship between the lipid peroxidation and antioxidants measures (Δ rate of change % (POMj-PLA)) following a weightlifting training session.

Variables	Acute Response	Delayed Response
Relationship between MDA and the enzymatic antioxidant system		
MDA—CAT	*r* = 0.6, *p* = 0.005	*r* = 0.6, *p* = 0.006
MDA—GPX	*r* = 0.5, *p* = 0.03	*r* = 0.5, *p* = 0.02
Relationship between MDA and the non-enzymatic antioxidant system		
MDA—UA	*r* = 0.7, *p* = 0.0008	*p* > 0.05
MDA—Tbil	*r* = 0.5, *p* = 0.04	*p* > 0.05

## Supplementary Materials

The following are available online at www.mdpi.com/2072-6643/9/8/819/s1. File S1: Receipt of Clinical trials.gov registry. Figure S1: Regression line for the significant relationships between the lipid peroxidation and antioxidants acute measures (Δ rate of change % (POMj-PLA)) following weightlifting training session, Figure S2: Regression line for the significant relationships between the lipid peroxidation and antioxidants delayed measures (Δ rate of change % (POMj-PLA)) following weightlifting training session.
