# Supplementary material for: Effects of Pomegranate Juice Supplementation on Oxidative Stress Biomarkers Following Weightlifting Exercise
Source: Nutrients. 2017 Jul 29;9(8):819. doi: 10.3390/nu9080819 (PMC5579613; doi:10.3390/nu9080819)
Supplement: Supplementary file 1 [file nutrients-09-00819-s001.zip › Figure S1-proof.pdf]

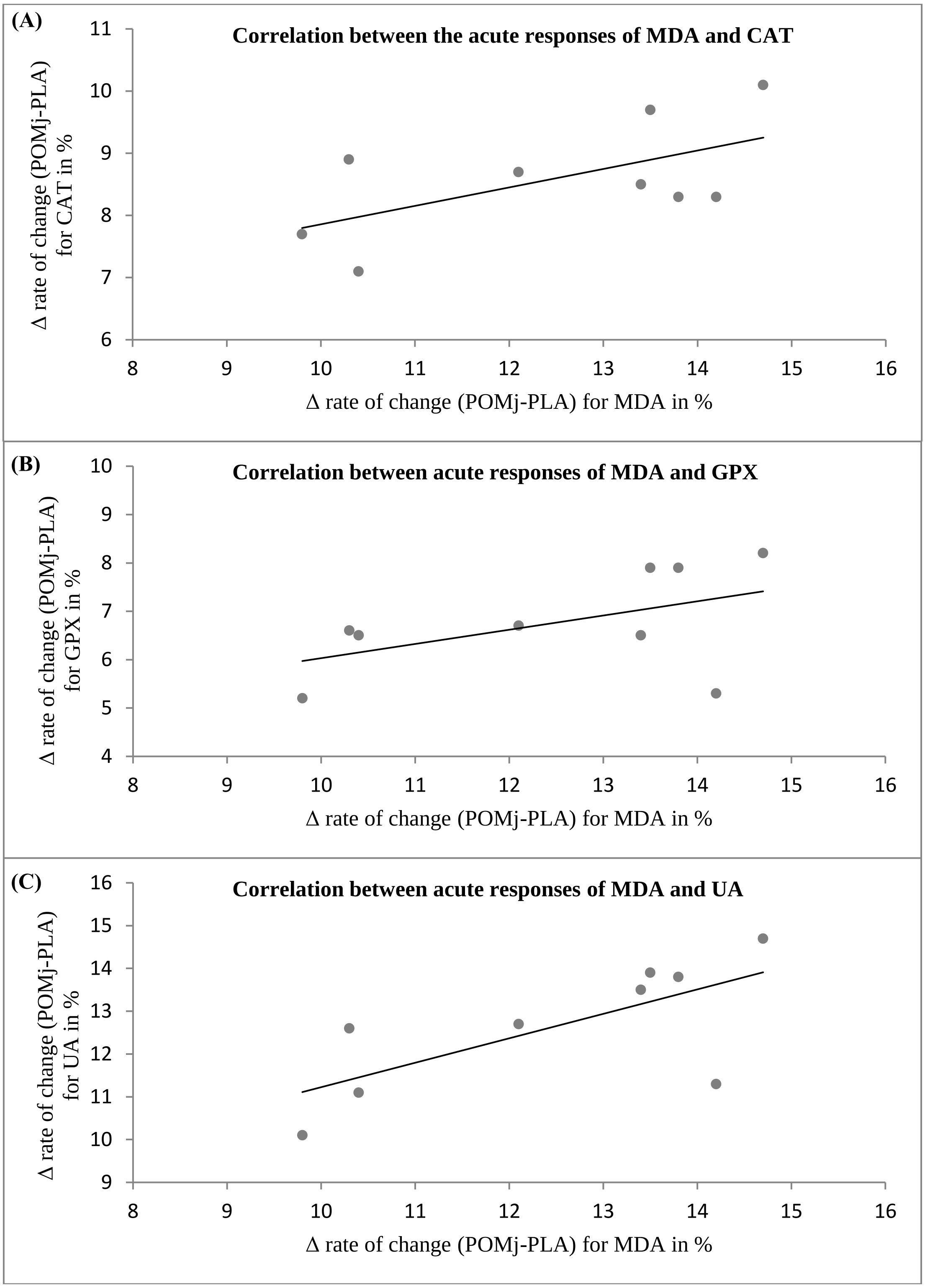

Figure S1: Regression line for the significant relationships between the lipid peroxidation and antioxidants acute measures (Δ rate of change % (POMj-PLA)) following weightlifting training session. (**A**). Correlation between the acute responses of MDA and CAT; (**B**). Correlation between acute responses of MDA and GPX; (**C**). Correlation between acute responses of MDA and UA.
